# Interferon Regulatory Factor-1 (IRF-1) Is Involved in the Induction of Phosphatidylserine Receptor (PSR) in Response to dsRNA Virus Infection and Contributes to Apoptotic Cell Clearance in CHSE-214 Cell

**DOI:** 10.3390/ijms151019281

**Published:** 2014-10-23

**Authors:** Hsin-Chia Kung, Øystein Evensen, Jiann-Ruey Hong, Chia-Yu Kuo, Chun-Hsi Tso, Fang-Huar Ngou, Ming-Wei Lu, Jen-Leih Wu

**Affiliations:** 1 Institute of Cellular and Organismic Biology, Academia Sinica, Taipei 11574, Taiwan; E-Mail: znga.hg@gmail.com; 2 Department of Basic Sciences and Aquatic Medicine, Norwegian University of Life Sciences, Oslo 0454, Norway; E-Mail: oystein.evensen@nmbu.no; 3 Institute of Biotechnology, National Cheng-Kung University, Tainan 70101, Taiwan; E-Mail: jrhong@mail.ncku.edu.tw; 4 Department of Aquaculture, National Taiwan Ocean University, Keelung 20224, Taiwan; E-Mails: key33_40@hotmail.com (C.-Y.K.); chunhsi.tso@hotmail.com (C.-H.T.); fanghuar@hotmail.com (F.-H.N.); 5 Center of Excellence for the Oceans, National Taiwan Ocean University, Keelung 20224, Taiwan

**Keywords:** phosphatidylserine receptor (PSR), interferon regulatory factor-1 (IRF-1), interferon (IFN), apoptosis

## Abstract

The phosphatidylserine receptor (PSR) recognizes a surface marker on apoptotic cells and initiates engulfment. This receptor is important for effective apoptotic cell clearance and maintains normal tissue homeostasis and regulation of the immune response. However, the regulation of PSR expression remains poorly understood. In this study, we determined that interferon regulatory factor-1 (IRF-1) was dramatically upregulated upon viral infection in the fish cell. We observed apoptosis in virus-infected cells and found that both PSR and IRF-1 increased simultaneously. Based on a bioinformatics promoter assay, IRF-1 binding sites were identified in the PSR promoter. Compared to normal viral infection, we found that PSR expression was delayed, viral replication was increased and virus-induced apoptosis was inhibited following IRF-1 suppression with morpholino oligonucleotides. A luciferase assay to analyze promoter activity revealed a decreasing trend after the deletion of the IRF-1 binding site on PSR promoter. The results of this study indicated that infectious pancreatic necrosis virus (IPNV) infection induced both the apoptotic and interferon (IFN) pathways, and IRF-1 was involved in regulating PSR expression to induce anti-viral effects. Therefore, this work suggests that PSR expression in salmonid cells during IPNV infection is activated when IRF-1 binds the PSR promoter. This is the first report to show the potential role of IRF-1 in triggering the induction of apoptotic cell clearance-related genes during viral infection and demonstrates the extensive crosstalk between the apoptotic and innate immune response pathways.

## 1. Introduction

The phosphatidylserine receptor (PSR) plays an important role in the clearance of apoptotic cells by recognizing phosphatidylserine (PS or PtdSer) on apoptotic cells and subsequently engulfing them. The PSR is the major receptor involved in apoptotic cell clearance during development [1] and is also associated with the phagocytosis of apoptotic cells in chronic pancreatitis [2]. PSR knockdown can result in the accumulation of large numbers of dead apoptotic cells in early embryos and can interfere with embryonic cell migration in zebrafish [3]. The protein size of mammalian PSR was previously reported to be approximately 48 kDa [4]. In addition to other species, zebrafish PSR (zfpsr) was cloned, and its nucleotide sequence was compared with corresponding sequences in mammals, such as humans (74%), and mice (72%) [3]. All macrophages, and indeed all phagocytes, recognize the marker PS, which is found on apoptotic cells. In normal live cells, PS is located in the inner cell membrane. When apoptosis is triggered by a stimulus, PS molecules are translocated from inside to the outer surface of the cell and act as signals for macrophages or other phagocytes to engulf the apoptotic cells [1,5]. PSR promotes phagocytes to take up tethered apoptotic cells with exposed PS molecules. Signals resulting from PSR-PS binding also leads to the secretion of immunosuppressive cytokines such as transforming growth factor beta (TGF-β) and possibly prostaglandin E2 (PGE2), interleukin-10 (IL-10), and platelet-activating factor (PAF), permitting PSR to act in an immunoregulatory capacity [6]. Therefore, PSR plays a dual role in both apoptotic cell clearance and anti-inflammation. Recently, it was confirmed that PSR serves as a membrane-associated receptor that regulates phagocytosis in immature macrophages and is actually expressed at the cell surface and regulates phagocytosis in immature monocyte-like activated human monocytic leukaemia (THP-1) cells [7]. However, the molecular and cellular mechanisms involved in the production of PSR as well as how this receptor exerts its effects in apoptotic cell clearance still remain unknown.

Apoptosis, or programmed cell death, is considered to be a physiological process that is involved in normal tissue turnover, which occurs during embryogenesis, aging, tumor regression, and as a consequence of immune effects, infection, or inflammation [8]. Cells undergoing apoptosis are eventually engulfed and digested by phagocytic cells [9]. Many studies have shown that host cells are induced to undergo apoptosis upon viral infection, and eliminated apoptotic cells are effectively cleared to maintain normal tissue homeostasis and regulation of the immune response. Based on the “waste disposal” hypothesis [10] and the “danger” hypothesis [11], a failure in apoptotic cell clearance can lead to the generation of plasma membrane-damaged cells (e.g., late apoptotic and necrotic cells) and the exposure of both immunostimulatory molecules and autoantigens to the immune system [10]. Based on our previous studies, infectious pancreatic necrosis virus (IPNV) infection can induce apoptosis through activating caspase-3, -8, -9 in salmonid and zebrafish cell lines, and apoptotic cells are engulfed by neighboring cells [12,13,14]. Therefore, the expression of PSR on these neighboring cells may promote the clearance of the apoptotic cells to eliminate plasma membrane-damaged cells. The Nakanishi group (2000) [15] previously studied apoptotic influenza A virus-infected cells and found that they are engulfed by macrophages; their results indicated that the apoptosis-dependent phagocytosis of virus-infected cells may lead to direct elimination of the viral pathogen. This study highlights the importance of apoptotic cell clearance to eliminate pathogens, either by neighboring cells or macrophages.

IPNV, which is the prototype virus of the family known as *Birnaviridae*, consists of a bi-segmented double-stranded RNA (dsRNA) molecule [16,17] and causes infectious pancreatic necrosis (IPN), a serious and contagious disease with high mortality, in salmonid fishes as well as a number of other non-salmonid fishes worldwide, resulting in severe losses in the aquaculture industry [18]. Due to the characteristic viral dsRNA intermediates, IPNV infection also mediates the interferon (IFN) pathway in salmonid cell lines [19,20,21,22,23]; activation of this pathway involves a general mechanism to induce the clearance of the viral infection. The IFN pathway triggers the synthesis of the IFN cytokines through janus kinase-signal transducer and activator of transcription (JAK-STAT) signal transduction. IFNs promote an antiviral state by inducing the myxovirus resistance (Mx) protein and other antiviral proteins [19]. This response represents an early host defense response and occurs prior to the non-specific immune response [19,24]. Most virus-infected cells are capable of synthesizing IFN-α/β, or the type I IFN system, in cell culture [24]; the type I IFN response plays a crucial role in the first line of defense against viral entry and infection through the secretion of IFN-α/β to protect uninfected cells from viral attack [20]. A variety of fish species, including salmonids, possess IFNs that have sequential similarity to mammalian type I IFNs [20,21,25].

IFN regulatory factors (IRFs), a family of transcription factors, play important roles in the IFN system during viral infections and other forms of cell stress [11,26]. In particular, interferon regulatory factor-1 (IRF-1) is indispensable for antiviral actions against certain viruses, including newcastle disease virus (NDV), encephalomyocarditis virus (EMCV), and Hepatitis C virus (HCV) [8,15,27], and this molecule is dramatically upregulated upon viral infection, treatment with double-stranded RNA, IFN stimulation, or treatment with other cytokines in a variety of cell types [27,28,29,30]. IRF-1 was the first IRF family member found to activate the *IFN-β* gene and is constitutively expressed in most cell types [28]. IRF-1 specifically binds to the upstream regulatory region of the human *IFN-β* gene and mediates virus-induced gene transcription [27], indicating that IRF-1 can exert its effects on genes by interacting with specific promoter regions. However, little is known about the mechanism by which IRF-1 activates the promoter of PSR upon viral infection. Given the role IRF-1 as a transcription factor, it has been presumed that increased IRF-1 expression is involved in the regulation of anti-viral gene expression, potentially including PSR.

To investigate the relationship between PSR and IRF-1 during viral infection, we wanted to investigate the structure of the PSR promoter. If the PSR promoter contained IRF-like binding sites for IRF-1, this would suggest that IRF-1 regulates PSR gene expression via promoter binding. Therefore, we were interested in understanding the transcriptional mechanism of PSR expression during viral infection. It is unknown whether IRF-1 plays a role in PSR induction during the viral infection. To expand our knowledge of PSR induction in salmonid cells during IPNV infection, we first confirmed the presence of apoptosis and the expression of PSR in infected cells. Simultaneously, we also found that IRF-1 increased constitutively during viral infection. We next cloned the promoter of PSR and studied PSR gene expression in response to different stimuli associated with viral infection or IFN treatment using either fluorescence or a luciferase reporter assay. Furthermore, we evaluated the effects of IRF-1 knockdown by deleting the PSR promoter and utilizing morpholino oligonucleotides. Our results suggested that IPNV infection induced both the apoptosis and the IFN pathways, and in particular IRF-1, which is involved in the latter pathway and is a regulator of PSR production that can exert anti-viral effects by promoting apoptotic cell clearance. Therefore, PSR expression in salmonid cells during IPNV infection might potentially be activated via IRF-1 binding to the PSR promoter. In the current study, we explored for the first time the potential role of IRFs in triggering the induction of PSR, an apoptotic cell clearance-associated gene, during viral infection, emphasizing the relevance of the relationship between apoptosis and the immune system.

## 2. Results and Discussion

### 2.1. Infectious Pancreatic Necrosis Virus (IPNV) Infection Induces Apoptosis and the Expression of Phosphatidylserine Receptor (PSR) in CHSE-214 Cells

**Figure 1:**
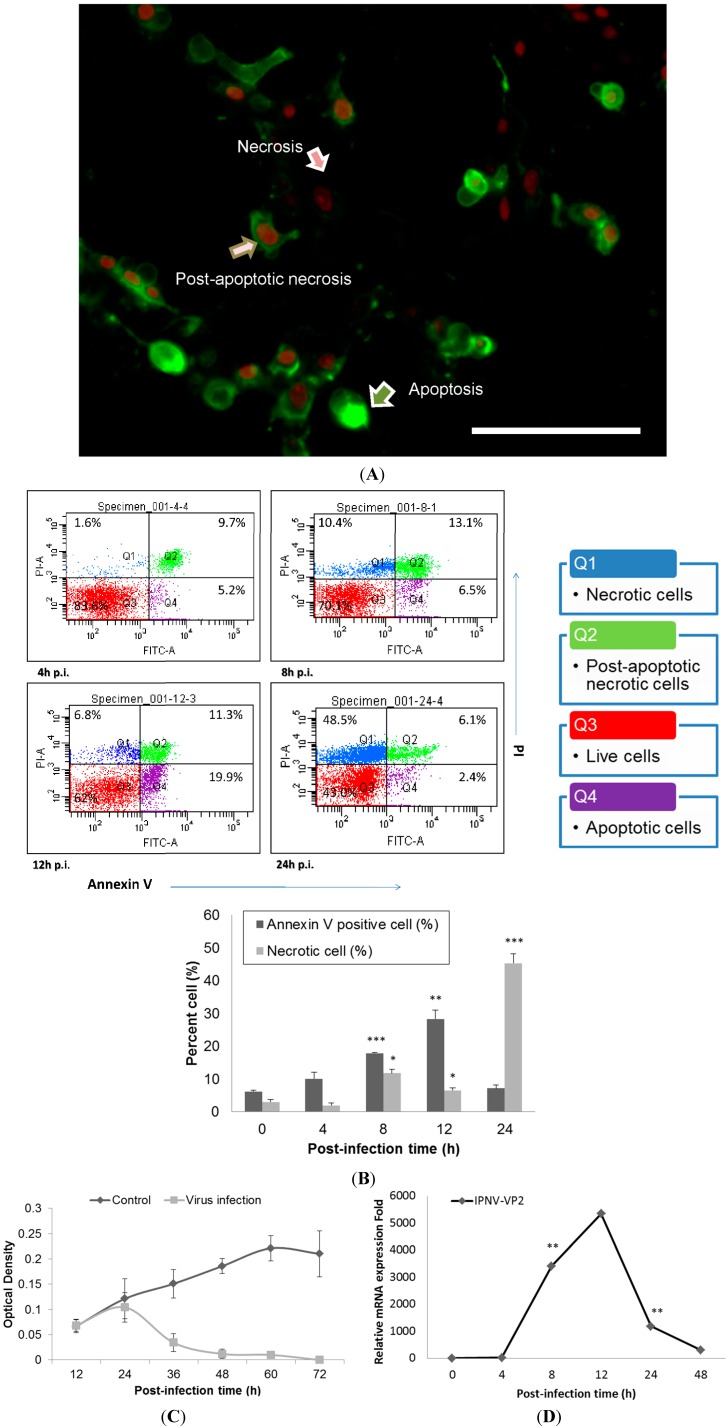
Apoptosis occurs in Infectious Pancreatic Necrosis Virus (IPNV)-infected CHSE-214 cells and viral replication reaches a peak at 12 h.p.i. (MOI = 1). (**A**) Annexin V and propidium iodide (PI) staining in infected CHSE-214 cells at 8 h.p.i.; three types of cells (pointed arrows) are delineated (scale bar = 100 μm). Annexin V-labeled cells indicated apoptosis, PI-labeled cells indicated necrosis, and double staining indicated post-apoptotic necrotic cells; (**B**) The infected cells were analyzed by flow cytometry for apoptosis, revealing a dynamic shift among the four types of cells. Double stained and PS-positive cells increased prior to 12 h.p.i., and then most cells became necrotic at 24 h.p.i. (* *p* < 0.05; ** *p* < 0.01; and *** *p* < 0.0001). All reported values have been corrected for background; (**C**) Cell viability was determined in triplicate by a WST-1 assay, which indicated that cell viability decreased after 24 h.p.i.; and (**D**) Real-time qPCR to determine the mRNA levels of viral VP2, a major capsid protein of IPNV. The results indicated high levels of replication among the infected cells that peaked and corresponded to the peak in apoptosis during IPNV infection. (** *p* < 0.01).

**Figure 2:**
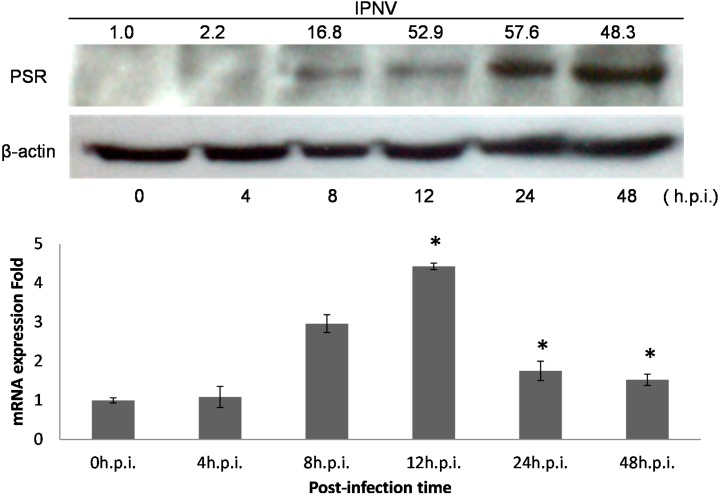
PSR expression is induced by IPNV infection in CHSE-214 cells. PSR expression was analyzed with polyclonal anti-PSR or anti-β-actin (loading control) antibodies by immunoblot (**upper panel**). Normalized density were calculated and listed above. Cells were infected with IPNV (MOI = 1), and proteins were isolated from cells at the times indicated. PSR (48 kDa) and β-actin (45 kDa) were analyzed at 0, 4, 8, 12, 24 and 48 h.p.i. in lanes **1**–**6**, respectively (0 h.p.i. as control). PSR proteins were detected starting at 8 h.p.i. and continuously increased until 48 h.p.i. The mRNA expression level was measured by real-time PCR in triplicate and normalized by β-actin. (**lower panel**) The data was expressed as the mean ± SD (* *p* < 0.05)

The cytopathic effect (CPE) of IPNV infection (MOI = 1) among CHSE-214 cells was observed at 8 h post-infection (h.p.i) and was found to increase dramatically as time increased; obvious cell death was observed between 12 and 48 h.p.i (Data not shown). IPNV infection induced apoptosis in CHSE-214 cells, and this was confirmed with double staining of annexin V and propidium iodide (PI) in the infected cells. Three types of the cells were identified at 8 h.p.i: Annexin V staining of exposed phospatidylserine (PS) indicated an apoptotic cell, PI in the nucleus indicated a necrotic cell, and dual staining indicated a post-apoptotic necrotic cell (Figure 1A). In the flow cytometry analysis, PS-positive cells consistently increased in number, reaching 28.3% (*p <* 0.01) of the total cells at 12 h.p.i., and then the majority of cells shifted to necrosis at 24 h.p.i. However, the CHSE-214 cell is very susceptible to the E1S strain IPNV, most of the cell showed CPE at 36 and 48 h.p.i. It is hard to collect the cells for fluorescence-activated cell sorting (FACS) assay. Hence, we only present the data from 0–24 h.p.i. only (Figure 1B). To evaluate the viability of infected CHSE-214 cells, WST-1 assays were performed in triplicate, revealing that viability decreased gradually after 24 h.p.i., which corresponded to the peak in necrosis (Figure 1B,C). Additionally, viral replication was analyzed by detecting the expression of viral capsid protein with real-time qPCR (Figure 1D) and immunoblotting (Data not shown), allowing us to monitor the progression of IPNV replication in infected salmonid cells. Real-time qPCR for viral VP2, a major capsid protein of IPNV, indicated that replication peaked at 12 h.p.i. (Figure 1D). Immunoblots confirmed the expression of PSR in apoptotic CHSE-214 cells during IPNV infection, and indicated that the PSR protein was induced by viral infection starting at 8 h.p.i. and remained consistently elevated until 48 h.p.i. (Figure 2).

### 2.2. The Analysis of the Interferon Regulatory Factor-1 (IRF-1) Binding Site on PSR Promoter

**Figure 3:**
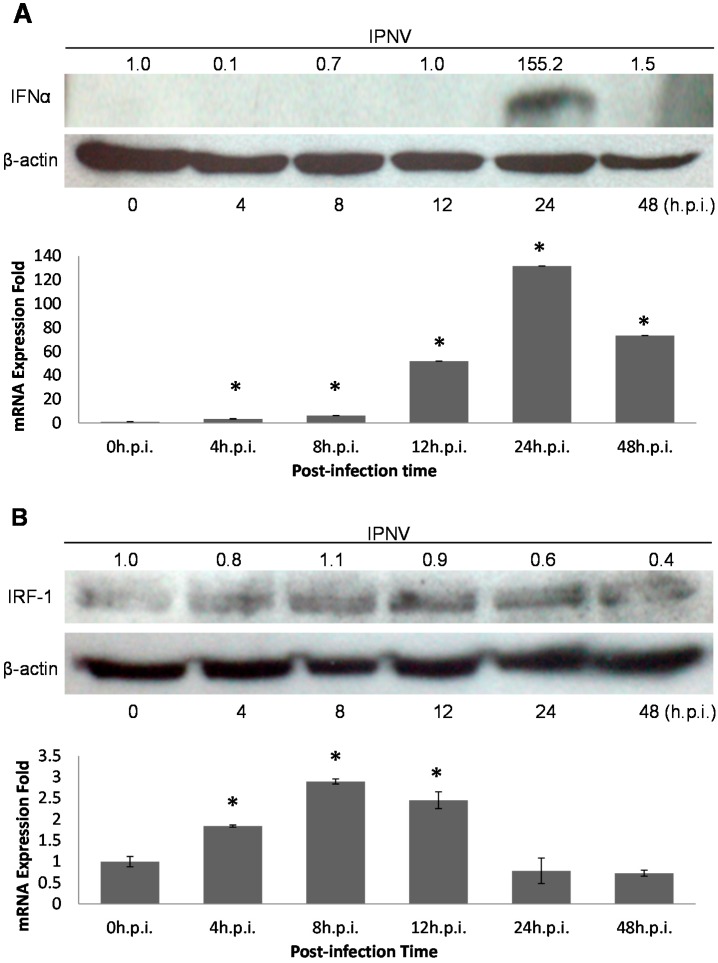
(**A**) rIFN-α induction after IPNV infection was analyzed with polyclonal anti-rIFN-α or anti-β-actin (loading control) antibodies by immunoblot (**upper panel**). Normalized density were calculated and listed above. The cells were infected with IPNV (MOI = 1), and proteins were isolated at the times indicated. rIFN-α (17 kDa) and β-actin (45 kDa) were analyzed at 0, 4, 8, 12, 24 and 48 h.p.i. in lanes **1**–**6** (0 h.p.i. as the control). rIFN-α proteins were detected at 24 h.p.i; **Lower panel** showed the relative mRNA expression to β-actin of IFN-α by using real-time PCR. The data was expressed as the mean ± SD (* *p* < 0.05); and (**B**) CHSE-214 cells were infected with IPNV (MOI = 1) and lysed at the indicated times for immunoblotting analysis with polyclonal anti-IRF-1 or anti-β-actin (loading control) antibodies (**upper panel**); Normalized density were calculated and listed above. The results indicated IRF-1 (51 kDa) was stably expressed in the infected cells from 0–48 h.p.i. (0 h.p.i. as the control). **Lower panel** showed the relative mRNA expression to β-actin of IRF by using real-time PCR. The data was expressed as the mean ± SD (* *p* < 0.05).

**Figure 4:**
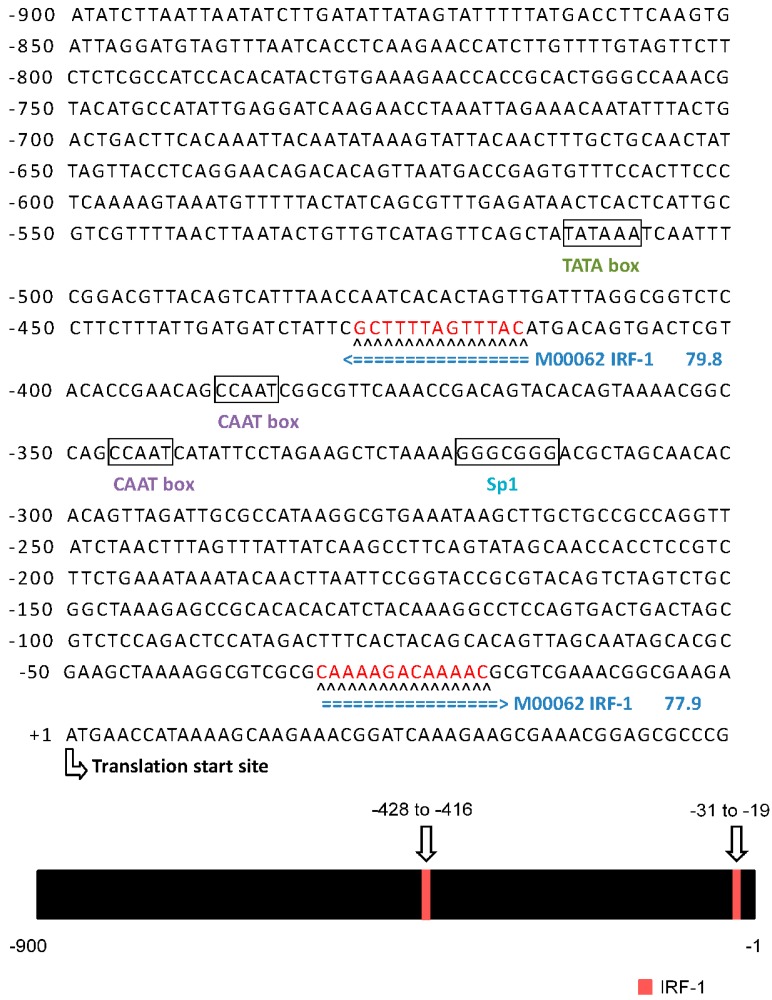
The sequence showed that two putative IRF-1 binding sites are located in the PSR promoter (**upper panel**). Putative transcription factor (TF) binding sites were identified with the TFSEARCH program (score setting = 75). There are two potential IRF-1 binding elements in the zebrafish 900-bp PSR promoter, and the consensus sequences (5'-G(A)AAA^G^/_C_^T^/_C_GAAA^G^/_C_^T^/_C_-3') of the IRF-1 transcription factor binding sites were located at positions −428 and −31 (red) relative to the putative *zf*PSR translation start site (bent arrow). The codes and flanked numbers depicted in blue color represent the TFSEARCH transcription factor identifier and Threshold score, respectively. The green and purple codes showed the region of Goldberg-Hogness (TATA) box and CAAT box, respectively. The **lower panel** shows a schematic representation of the *PSR* gene promoter, and the positions of putative IRF-1 binding sites on the fragment are indicated by arrows.

Induction of the interferon (IFN) pathway is a general mechanism to combat viral infection. To confirm that the rIFN-α pathway is activated by IPNV dsRNA, rIFN-α expression patterns were analyzed by real-time qPCR and immunoblotting (Figure 3A). Both tests implied that the *rIFN-α* gene was induced during IPNV infection. A previous report suggested that IRF-1 is involved in the rIFN-α downstream pathway and is induced by IPNV infection; therefore, we sought to confirm the presence of IRF-1 binding sites in the PSR promoter. Because the sequence of the salmonid PSR promoter is unknown, we selected the zebrafish PSR sequence to analyze the structure of the PSR promoter. Putative transcription factor (TF) binding sites were identified with the TFSEARCH program (score setting = 75). We found that there are two IRF-1 binding elements within the 900-bp sequence upstream of the putative transcription start site of *zf*PSR, and the consensus sequences (5'-G(A)AAA^G^/_C_^T^/_C_GAAA^G^/_C_^T^/_C_-3') of the IRF-1 transcription factor binding sites were located at positions −428 and −31 relative to the putative *zf*PSR transcription start site (Figure 4). We also demonstrated that IRF-1 proteins are constitutively expressed in cells during IPNV infection (Figure 3B).

### 2.3. Effects of PSR Promoter Regulation on rIFN-α or IPNV Induction

**Figure 5:**
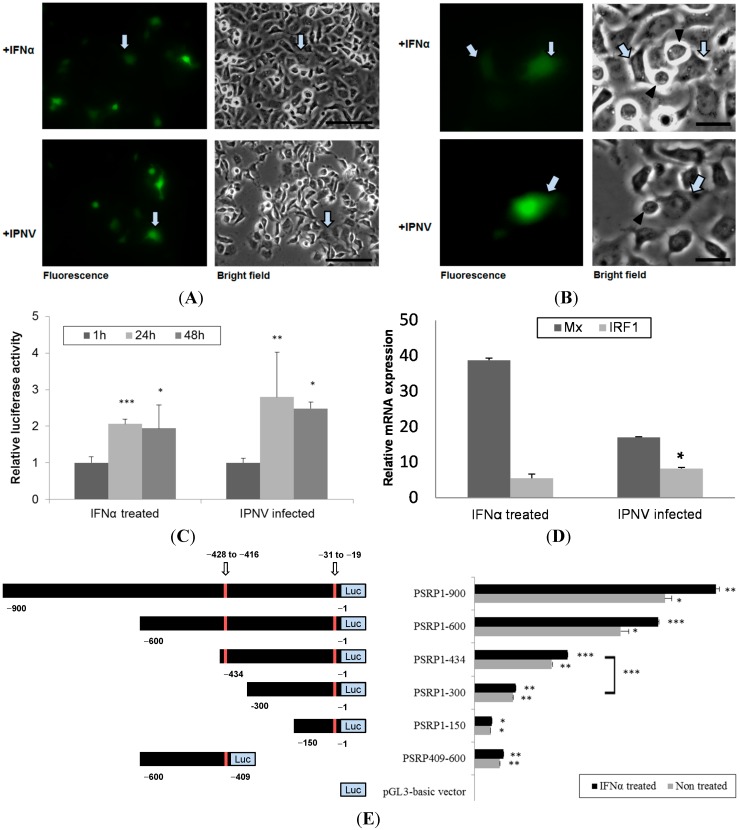
The PSR promoter can be induced by rIFN-α or IPNV in CHSE-214 cells. (**A**) CHSE-214 cells were transfected with the pPSRP:EGFP construct, stimulated with either rIFN-α (0.5 μg/mL) or IPNV (MOI = 1) for 24 h, and then visualized by fluorescent microscopy. The PSR promoter was activated by the stimuli (fluorescent green pointed by arrows; scale bar = 100 μm); (**B**) PSR promoter-activated cells (green) had the potential to engulf apoptotic cells (pointed triangle) in both rIFN-α or IPNV-stimulated cells (scale bar = 25 μm); (**C**) CHSE-214 cells were transfected with the *pGL3-basic-PSRP* construct, stimulated with either rIFN-α or IPNV for 24 and 48 h, and then evaluated with a luciferase assay. PSR promoter activity was expressed as fold induction of relative luciferase activity (RLU) compared to the promoterless vector and then compared with the values at 1 h. The data indicated that the PSR promoter was activated by the stimuli (* *p* < 0.05; ** *p* < 0.01; and *** *p* < 0.0001); (**D**) Mx and IRF-1 were induced in pPSRP:EGFP expressing cells as determinedby real-time qPCR. Relative expression is compared to beta-actin expression as internal control (* *p* < 0.05); and (**E**) Promoter activity of 900-bp PSR promoter and IRF-1-deleted PSR promoter. The **left panel** shows a schematic representation of the *PSR* gene promoter constructs. Full-length PSR promoter (−900 to −1) as well as various deleted fragments were inserted into the pGL3-basic plasmid with luciferase (*Luc*). Putative IRF-1 binding sites in the fragment are indicated by arrows and marked in red. PSR promoter activity in response to rIFN-α (0.5 μg/mL) was assessed 48 h after the addition of the cytokine and expressed as fold induction of relative luciferase activity (RLU) compared to the promoterless vector. Cells exposed to basal medium without the cytokine were used as a control. Luciferase assays (right panel) were performed in triplicate, and the data are expressed as the mean ± SD (* *p* < 0.05; ** *p* < 0.01; and *** *p* < 0.0001).

To evaluate PSR promoter activity, we cloned the 900-bp PSR promoter into either a promoterless enhanced green fluorescent protein (EGFP) reporter gene vector or a promoterless luciferase reporter gene vector. The pPSRP:EGFP construct was transfected into CHSE-214 cells by electroporation and then induced with rIFN-α or IPNV for 24 h. rIFN-α exhibited approximately 60% sequence identity to salmon IFN for the region lying between amino acids 125 and 136 [31]. A test for rIFN-α induction of the PSR promoter construct was performed and indicated that 0.5 μg/mL was enough concentration for induction (data not shown). RNA viruses induce IFN production via their dsRNA intermediates; thus, the effects of viral infection on the PSR promoter were examined. For this purpose, the aquatic birnavirus IPNV was used, and an MOI of 1 was able to induce the PSR promoter by 2- to 3-fold at 24 and 48 h after infection (Figure 5C). With fluorescence microscopy, we observed PSR expression in PSR promoter-activated cells following induction with rIFN-α or IPNV (Figure 5A). Interestingly, we observed that PSR promoter-activated cells might be surrounded by the apoptotic cells, indicating that the PSR promoter-activated cells had the potential to engulf the apoptotic cells following rIFN-α or IPNV stimulation (Figure 5B). These results corresponded with our previous study [32] but also demonstrated that neighboring cells, which would engulf apoptotic cells, can express PSR to recognize PS on the surface of the apoptotic cells [32]. To evaluate the induction of the type I IFN pathway in activated cells, we harvested EGFP-activated cells and subjected them to real-time qPCR analysis to analyze the expression patterns of two downstream genes, Mx and IRF-1, which are both involved in the type I IFN pathway. We found that both of these genes were induced in pPSRP:EGFP expressing cells (Figure 5D), suggesting that type I IFN pathway is activated in response to viral infection and IRF-1 may possibly regulate the expression of PSR.

Additionally, CHSE-214 cells were transfected with both the full-length (900 bp) PSR promoter and the truncated IRF-1 binding region in the *pGL3-basic-PSRP* construct. The following upstream PSR gene constructs were synthesized: pPSRP1-900, pPSRP1-600, and pPSRP1-434, which contain two IRF-1 binding sites; and pPSRP1-300, pPSRP1-150, and pPSRP409-600, which contain one IRF-1 binding site (Figure 5E, the left panel shows a schematic representation of the *PSR* gene promoter constructs). After transfection via electroporation, the cells were stimulated with either rIFN-α or IPNV and then evaluated with the luciferase assay. Full-length *PSR* promoter activity was higher in terms of relative luciferase activity (RLU) compared to the promoterless vector. The results of this luciferase assay indicated that the 900-bp *PSR* promoter was activated by the stimuli at 24 and 48 h.p.i. (Figure 5C). In the promoter deletion assay, we first analyzed the luciferase activity of pPSRP1-900, pPSRP1-600, pPSRP1-300, and pPSRP1-150 and found that both PSRP300-600 deletions resulted in reduced activity (Data not shown). This implied that important transcription factor binding sites were located in this region, and indeed, we found that it contains a putative IRF-1 binding element. Therefore, we further constructed various deleted fragments with one or two IRF-1 binding elements and analyzed them in the luciferase assay. IRF-1-deleted *PSR* promoter activity in response to rIFN-α (0.5 μg/mL) induction was assessed 48 h after the addition of the cytokine and exhibited greater induction in terms of relative luciferase activity (RLU) compared to the promoterless vector. Cells exposed to basal medium without the cytokine were used as a control. Figure 5E displays the luciferase activity of several different deleted constructs for rIFN-α-stimulated or untreated CHSE-214 cells, and all promoter constructs show increasing luciferase activity at 48 h after rIFN-α treatment. In this assay, induction was highest for those constructs that contained two IRF-1 binding sites, including pPSRP1-900, pPSRP1-600 and pPSRP1-434; and lower for pPSRP1-300, pPSRP1-150 and pPSRP409-600, which had just one IRF-1 binding element. Accordingly, these results indicated that PSRP300-434 was most important for PSR induction in the cells and this region potentially contained an IRF-1 binding site. The full-length 900-bp PSR promoter was induced by more than 20-fold, whereas the minimal 150-bp PSR promoter was induced by only 1.8-fold at 48 h after rIFN-α treatment. In addition, pPSRP409-600 exhibited expression that was similar to the minimal promoter construct pPSRP1-150. The expression of pPSRP409-600 was significant but not much higher than pPSRP1-150, which may indicated that the IRF-1-containing PSR promoter is important for *PSR* gene expression.

### 2.4. Effects of IRF-1 Knockdown on CHSE-214 Cells during IPNV Infection

**Figure 6:**
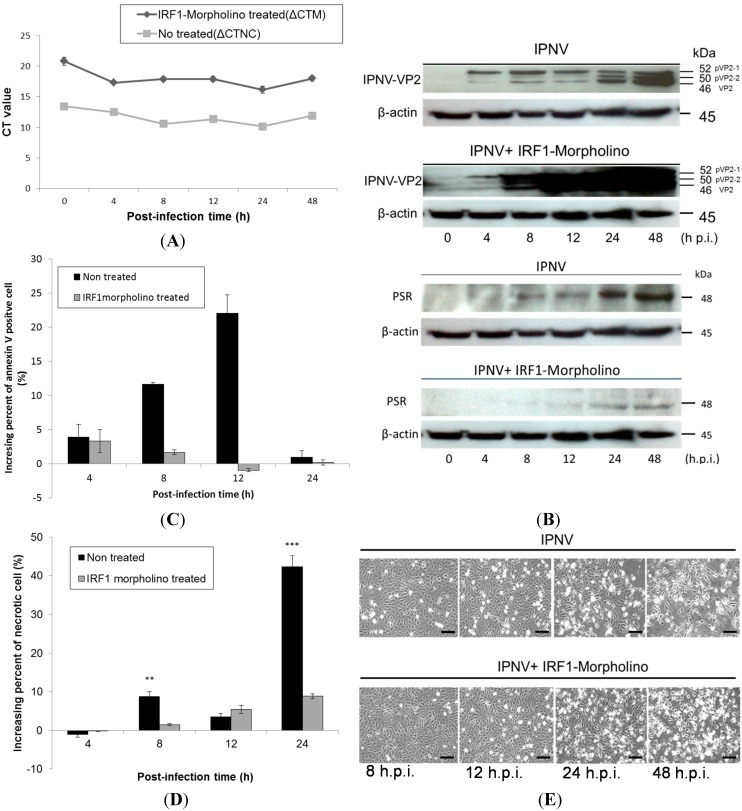
Effects of IRF-1 knockdown on CHSE-214 cells during IPNV infection. (**A**) Real-time qPCR was used to determine the mRNA levels of IRF-1 with or without IRF-1 morpholino treatment following IPNV infection (MOI = 1). Expression were normalized to the internal control (β-actin) and is shown as the Δ*C*_t_ value. The results indicated that IRF-1 mRNA expression was inhibited by approximately 38% with the morpholino; (**B**) Immunoblots of PSR or IPNV-VP2 expressionin IPNV-infected CHSE-214 cells (MOI = 1); The **upper panels** of (**B**) show the expression without IRF-1 morpholino treatment during infection, and the **lower panels** show the expression with IRF-1 morpholino treatment (10 μM, 18 h incubation) during infection. Lanes **1**–**6** correspond to 0, 4, 8, 12, 24 and 48 h.p.i., respectively (0 h as positive control, β-actin as internal control). PSR expression exhibited a delay in IRF-1 knockdown CHSE-214 cells compared to the control group (non-morpholino treated), whereas the IPNV viral protein VP2 dramatically increased in the same cells during IPNV infection; (**C**,**D**) Flow cytometry to analyze apoptosis or necrosis in CHSE-214 cells during IPNV infection. The data indicated that PS-positive cells and necrotic cells decreased in IRF-1 knockdown CHSE-214 cells (** *p* < 0.01; and *** *p* < 0.0001); and (**E**) Cell death was inhibited in IRF-1 knockdown CHSE-214 cells (**lower panel**), compared with control (**upper panel**) after IPNV infection (scale bar = 100 μm).

To clarify the relationship between IRF-1 and PSR, IRF-1 was knocked down with morpholino oligos during IPNV infection. We delivered the salmon IRF-1 morpholino (10 μM) into CHSE-214 cells first by electroporation and then incubated the transfected cells for 18 h before IPNV infection (MOI = 1). The morpholino oligos were conjugated with fluorescein isothiocyanate (FITC) to enable easy observation of the delivery efficiency by fluorescence microscopy (Data not shown). After the IRF-1 morpholino was delivered into the cells, its inhibitory effects on IRF-1 were confirmed by real-time qPCR (Figure 6A) and immunoblotting. Both tests showed the reduction of IRF-1 mRNA and protein expression in IRF-1 morpholino-treated CHSE-214 cells during IPNV infection. Figure 6A shows IRF-1 mRNA expression with or without IRF-1 morpholino treatment following IPNV infection in terms of Δ*C*_t_ value, which was normalized with the internal control (β-actin). The results indicated that IRF-1 mRNA expression was inhibited by approximately 38% with morpholino treatment. We next examined PSR expression in IRF-1 knockdown CHSE-214 cells during IPNV infection and found that PSR expression was delayed in IRF-1 knockdown cells during viral infection (Figure 6B, β-actin as internal control). All samples were probed with anti-PSR antibodies, and morpholino-treated samples exhibited delayed PSR expression until 24 h.p.i., whereas non-morpholino-treated samples expressed PSR earlier at 8 h.p.i. We also wanted to investigate viral replication under IRF-1-inhibited conditions. To analyze viral replication in IRF-1 morpholino-treated or untreated infected CHSE-214 cells, the expression of the major capsid protein VP2 of IPNV was evaluated by immunoblot. The viral VP2 protein was detected as early as 4 h.p.i. in infected CHSE-214 cells, and the expression of VP2 protein gradually transitioned from the precursor form, pVP2-1 (52 kDa), and intermediate form, pVP2-2 (50 kDa), to the mature form VP2 (46 kDa) between 8 and 48 h.p.i. (Figure 6B, lanes 3–6) in the infected cells. The morpholino-treated samples exhibited dramatically increasing expression of the VP2 protein between 8 and 48 h.p.i. in IRF-1 knockdown cells (Figure 6B, lower panel). Additionally, expression of the viral protein increased constitutively from 4–48 h after viral infection in non-morpholino treated cells (Figure 6B, upper panel).

**Figure 7:**
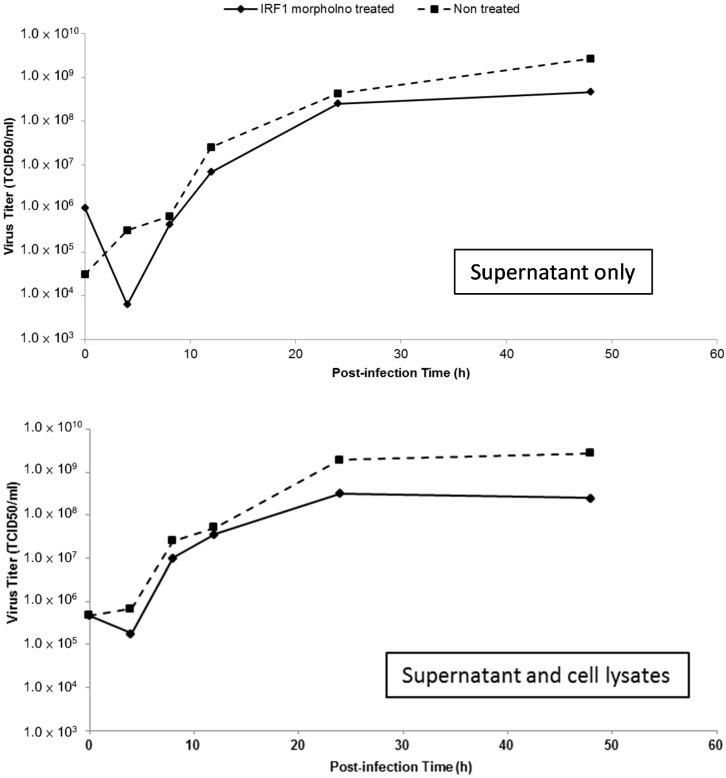
Viral titers in IRF-1 knockdown CHSE-214 cells during IPNV infection. After IPNV infection, supernatants and cell lysates were harvested and titrated for 50% tissue culture infective dose (TCID_50_).

The variation in the percentages of apoptotic and necrotic cells among IRF-1 morpholino-treated or untreated CHSE-214 cells was evaluated by flow cytometry during IPNV infection. The results showed that PS-positive cells (annexin V-stained) decreased in IRF-1 knockdown cells between 4 and 24 h.p.i. during IPNV infection (Figure 6C), and necrotic cells were also decreased in IRF-1 knockdown CHSE-214 cells at 8 and 24 h.p.i. (Figure 6D). The changes in morphology and mortality in IRF-1 morpholino-treated cells (Figure 6E, lower panel) were less severe than in non-morpholino cells (Figure 6E, upper panel) during IPNV infection, and these results hint that IRF-1 knockdown corresponds to reduced apoptotic cell death and necrotic cell death. Therefore, cell death was inhibited in IRF-1 knockdown CHSE-214 cells after IPNV infection, and IRF-1 might be required for the inhibition of viral replication. To evaluate viral titers in IRF-1 morpholino-treated CHSE-214 cells, we also determined the 50% tissue culture infective dose (TCID_50_) but found that no increase in the number of whole viral particles was detected after infection of the IRF-1 morpholino-treated cells compared to the non-morpholino-treated samples (Figure 7). This result implies that although viral replication is boosted in the IRF-1 morpholino-treated CHSE-214 cells, whole viral particles might not fully assemble to produce more viral particles.

**Figure 8:**
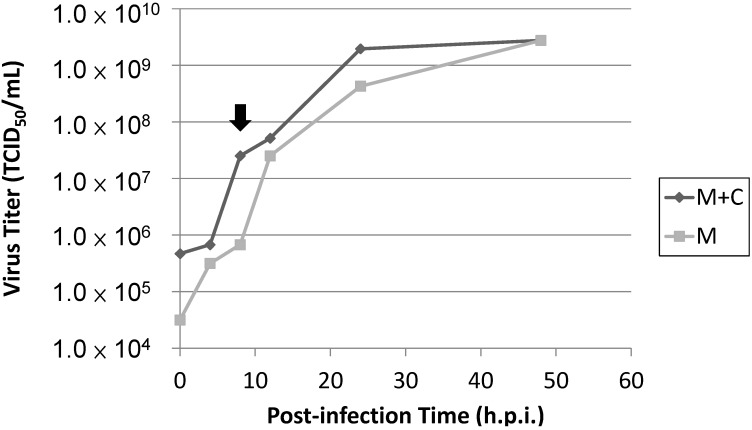
TCID_50_ for viral titers in IPNV-infected cells. Totl viral particles were harvested from the medius as well as the cell pellets (M + C). The total number of viral particles was higher that the medius—Only viral particles (M) at each time point during the infection, while viral yields for both were equal at 48 h.p.i. The difference between (M + C) and (M) was due to the viral particles inside the nfected cells. Based on these results, viral replication rate reached a maximus inside the cells at 8 h.p.i. (point arrow). The time period between 8 and 10 h.p.i. represents the maximum total synthesis of virus-specific RNA.

Several defense mechanisms to combat viral infection have been elucidated among the innate immune responses in host cells. In general, a variety of mechanisms have evolved. One antiviral defense mechanism is the apoptosis of host cells to facilitate voluntary cell death and prevent viral replication and spreading. Additionally, the host cell can also induce protective antiviral immune responses via the release of interferon (IFN), a cytokine that can enhance the antiviral response and stimulate the immune response to limit the extent of viral infection. In this study, we used IPNV, an important fish pathogen belonging to the family *Birnaviridae*, to infect salmonid cells and investigated cellular defenses in host cells upon viral infection. IPNV infection primarily causes symptoms of cellular necrosis in fishes, but a previous study showed that infection can induce atypical apoptosis preceding necrosis in a salmonid fish cell line [33]. Specifically, this study demonstrated that infected cells first appeared apoptotic and subsequently switched to secondary necrosis following pre-necrosis in salmonid cells during IPNV infection. The present findings indicated that IPNV infection caused cell death in salmonid cells and that apoptosis preceded necrosis (Figure 1A,B). We observed that the CPE of the cells severely increased as the infection progressed (data not shown), and the viability of the infected cells dramatically decreased after 24 h.p.i. (Figure 1C), corresponding to the peak in necrosis in the infected cells (Figure 1B). Additionally, the presence of IPNV viral titers in the infected cells as detected by the TCID_50_ assay indicated that the viral replication rate reached a peak at 8 h.p.i during infection (Figure 8), and these data corresponded to previously published studies that indicate the total synthesis of virus-specific RNA reaches its maximum level between 8 and 10 h.p.i. [34].

Elimination and clearance of virus-infected apoptotic cells is very important for host cell homeostasis and effective anti-viral action. Strikingly, previous results demonstrated that failure to engulf and remove these nearly dead cells may result in the survival of some cells, resembling the withdrawal from complete apoptosis. While such a reversal may not be of consequence in the context of the short lifespan of *C. elegans*, a similar reversal in fate for a human cell could be dangerous, as it could prevent the quick clearance of apoptotic cells [9]. Phagocytosis is a key process involved in eliminating virus-infected dying cells from an organism. Under normal physiological conditions, dying cells and pathogens are rapidly detected and removed by professional phagocytes such as macrophages and dendritic cells (DCs). In most cases, specific receptors on target cells are recognized and bound by phagocytes, triggering the intracellular signaling events required for phagocytosis [10,35]. Our previous study demonstrated that in addition to macrophages (professional phagocytes), which are capable of phagocytosing apoptotic cells, neighboring cells (non-professional phagocyte) were also capable of engulfing apoptotic salmonid cells during IPNV infection [32]. Moreover, several research papers have determined that the phosphatidylserine receptor (PSR) plays a crucial role in the clearance of apoptotic cells. PSR serves as a membrane-associated receptor that regulates phagocytosis, and this has been confirmed in immature macrophages; additionally, this receptor has other functions when expressed in the cytosol and nuclei of mature macrophage-like cells [7]. Therefore, we hypothesized that IPNV-induced apoptotic salmonid cells could be engulfed by neighboring cells via PSR binding with phosphatidylserine (PS). Although the mechanism of PSR induction is not yet clear, the type I IFN response is a general mechanism that is induced by viral infection, and thus we proposed a model suggesting a possible connection between them.

IFNs play an important role in innate immunity by inhibiting the replication and spread of pathogens, such as viruses, bacteria and parasites. Additionally, IFNs are key modulators of the immune response and exert anti-proliferative effects in some cell types. As a result, IFNs are used clinically to treat certain viral infections as well as some cancers and auto-immune diseases [11]. IFNs are commonly grouped into two categories: Type I IFNs, also known as viral IFNs, including IFN-α and IFN-β, which are induced by viral infection, and type II IFN, also known as immune IFN or IFN-γ, which is induced by mitogenic or antigenic stimuli [19,32]. Robertsen B *et al.* (2003) [31] determined that IFN was induced by poly I:C treatment in the head kidneys of Atlantic salmon, and salmonid IFN-α1 promoted the induction of the antiviral protein Mx in CHSE-214 cells. This suggested that the type I IFN system existed in fishes as well as in mammals. We determined that the IFN pathway was induced by IPNV dsRNA, and PSR expression was also elevated upon IPNV infection in our system (Figure 2 and Figure 3A). Interestingly, rIFN-α proteins were induced early during IPNV infection but were not detected after 24 h.p.i. (Figure 3A). This suggests that IPNV may be able to inhibit IFN signaling in fish cells and has evolved mechanisms to overcome IFN responses [32,36,37]. Therefore, the virus may have its own strategies to combat cellular defenses, such as the secretion of proteins that can control cellular apoptotic processing by regulating the expression of related genes to promote a suitable environment for viral replication in host cells.

Cells secrete type I IFNs in response to viral infection following the elevated activity of various transcription factors (TFs) including the interferon regulatory factor (IRF) family [28,38], which regulates innate response gene transcription as transcriptional activators or repressors depending on the target gene and their binding partners [39]. Nine members of the mammalian IRF family (IRFs 1–9) are known, and in particular, mammalian IRF-1, IRF-3 and IRF-7, are known to regulate the expression of type I IFNs, which are the cytokines involved in antiviral defense in response to virus-mediated signaling [11,26,36]. During viral infection, IRF-3 plays an early role in inducing the transcription of IFN-β, which in turn induces the expression of IRF-7 and then promotes the transcription of members of the IFN-α gene family, thereby creating a positive-feedback loop that boosts the host antiviral response [38]. IRF-1 is also induced by IFNs after IRF-3 and IRF-7 induction and hence it is located more downstream than IRF-3 and IRF-7 in the type I IFN signaling pathway [19,26,36,38,40]. Based on this, we analyzed the promoter of PSR and found that there were IRF-1 binding elements located 900 bp upstream of the *PSR* gene that might constitute the specific transcription factor binding region because the repetitive sequence that might be an enhancer region of the *PSR* gene was located farther upstream. The putative IRF-1 binding sites in the PSR promoter suggested that IRF-1 can regulate *PSR* gene expression via promoter binding, during viral infection (Figure 2 and Figure 4). Some variation between PSR protein levels and mRNA levels (data not shown) may be explained by the insufficient specificity of the primers used for *PSR* gene detection, which were designed from the zebrafish sequence because the sequence of salmon PSR is unknown.

After locating the PSR promoter, which promotes *PSR* gene expression during IPNV infection, we tried to elucidate the transcriptional mechanism of PSR by analyzing the TF binding sites in the promoter. Because the sequence of the salmonid PSR promoter is unknown, we selected the zebrafish sequence despite the differences between the two genes. Zebrafish IRF-1 displayed 40%–84% similarity to other fishes and 35%–39% similarity to other vertebrate IRF-1 sequences. We were still able to identify putative IRF-1 binding sites in the zebrafish PSR promoter. In addition, both PSR and IRF-1 were elevated after IPNV infection independent of the immunoblot results (Figure 2 and Figure 3) or the qPCR results. As the zebrafish PSR promoter contained putative IRF-1-binding sites, we tested whether rIFN-α or IPNV could induce the PSR promoter. We cloned the 900-bp promoter of zebrafish PSR and transfected it into salmonid cells following by IPNV infection or rIFN-α treatment to induce promoter activity. The data in Figure 5A–C indicated that the PSR promoter was activated either by IPNV infection or rIFN-α treatment. Furthermore, we deleted the full-length PSR promoter and then observed that luciferase activity decreased with decreasing promoter length.

To clarify the relationship between PSR and IRF-1, we used an IRF-1-deleted PSR promoter construct and translation-blocking IRF-1 morpholinos to reduce the expression levels of IRF-1 in salmonid cells during viral infection and observed the effects after knockdown. When the IRF-1-deleted PSR gene promoter was analyzed, a markable decrease in the promoter activity was observed when the IRF-1 binding site from −434 to −300 was deleted, and pPSRP409-600 showed similar effects compared to the minimal promoter pPSRP1-150 but exhibited slightly higher promoter activity (Figure 5E). These results suggested that the IRF-1 binding site in the PSR promoter is crucial for *PSR* gene expression. Additionally, we determined that the expression of IRF-1 was suppressed during IPNV infection (Figure 6A) after salmon IRF-1 morpholino oligos were transferred into the salmonid cells. Simultaneously, the PSR expression was delayed until 24 h.p.i., whereas the non-morpholino treated samples expressed PSR earlier at 8 h.p.i. (Figure 6B), and viral protein replication dramatically increased IRF-1 knockdown CHSE-214 cells (Figure 6C). Therefore, it can be assumed that PSR expression in salmonid cells during IPNV infection is induced through IRF-1 regulation. The variations in the percentages of virus-induced apoptosis and necrosis in IRF-1 knockdown CHSE-214 cells were quantitated with flow cytometry. The results indicated that the number of PS-positive cells decreased markedly in IRF-1 knockdown cells during IPNV infection (Figure 6D), and the number of necrotic cells also decreased in IRF-1 knockdown CHSE-214 cells at 8 and 24 h.p.i. (Figure 6E). However, the morphology and mortality of IRF-1 morpholino-treated cells changed, and the CPE was less severe compared to non-morpholino cells during IPNV infection (Figure 6F).These results indicated that IRF-1 knockdown corresponds to reduced apoptotic cell death and necrotic cell death. Therefore, cell death is inhibited in IRF-1 knockdown CHSE-214 cells after IPNV infection, whereas viral replication is boosted, and it can be suggested that IRF-1 is required to inhibit viral replication. Conversely, the results obtained with the IRF-1 morpholino demonstrate that IRF-1 is required for virus-induced apoptosis, which corresponds with the data of MT Stang *et al.* (2007) [41], who showed that IRF-1 can induce ligand-independent FADD/caspase-8-mediated apoptosis in breast cancer cells despite the fact that they used IFNγ as an inducer. Thus, IRF-1 is required for apoptosis induction. In general, IRF-1 activity does not lead to cell death, whereas it promotes apoptosis under certain physiological and pathological conditions (e.g., DNA damage or viral stimuli) [26,40]. Overall, IRF-1 knockdown in infected cells will inhibit the IPNV-induced cytopathic effect as well as IPNV-induced apoptotic and necrotic events, thus increasing IPNV viral protein yields. Strikingly, viral titers measured by TCID_50_ in IRF-1 knockdown cells did not reveal increased numbers of whole replicated viral particles in the culture medium (Figure 7). However, we could not provide a full answer to this phenomenon in this study. This interesting question requires further experimentation in future research.

## 3. Experimental Section

### 3.1. Cells, Virus and Infection

Chinook salmon embryo cells (CHSE-214) obtained from the American Type Culture Collection (ATCC, Manassas, VA, USA) were grown at 18 °C in Eagle’s minimum essential medium (MEM, Sigma–Aldrich, St. Louis, MO, USA) supplemented with 10% fetal bovine serum (FBS, invitrogen, Auckland, NZ, USA), 100 IU penicillin, and 0.1 mg/mL streptomycin (Gibco, Carlsbad, CA, USA). The aquabirnavirus used in this study, IPNV E1-S, a member of the Ab strain of IPNV, was isolated from a Japanese eel (*Anguilla japonica*) in Taiwan [42]. It was propagated in CHSE-214 cells at a multiplicity of infection (MOI) of 0.01, and the infected cultures were incubated at 18 °C until extensive cytopathic effect (CPE) was observed. When the CPE was observed after 5 days of post-infection (p.i.), the culture supernatants were harvested and stored at −80 °C until challenge. The supernatant viral titer was determined using an infectivity assay and was calculated as 1 × 10^8^ TCID_50_/mL. A multiplicity of infection (MOI) of 1 was used for all live IPNV experiments.

### 3.2. Prediction of Transcription Factor (TF) Binding Sites in the Zebrafish PSR Promoter

Transcription factor binding sites in the zebrafish PSR promoter were predicted with the TFSEARCH version 1.3 software (Parallel Application TRC Laboratory, RWCP, Gokasho, Japan) [43]. The salmon PSR promoter sequence is not available, and therefore the zebrafish promoter was used for further studies. This sequence was obtained from the Sanger Institute zebrafish genomic database (sequence from [44]; Ensembl Gene ID: ENSDARG-00000034358; Figure 4).

### 3.3. Plasmids for Fluorescence and Luciferase Assays

Genomic DNA was extracted from ZFLT cells (zebrafish liver cell line) using the Genomic DNA Mini Kit (Geneaid, Taipei, Taiwan). The 5' flanking region of the PSR gene was PCR-amplified with Platinum^®^ *Taq* DNA polymerase HiFi (Invitrogen, Carlsbad, CA, USA) using a PSR promoter forward primer and a reverse primer (sequence from Ensembl Gene ID: ENSDARG-00000034358). PCR conditions consisted of 94 °C for 2 min followed by 35 cycles of 94 °C for 30 s, 52 °C for 1 min, and 68 °C for 1 min, with a final extension at 72 °C for 10 min. The expected 900 bp fragment was obtained. Gel-purified fragments (Qiaquick Gel Extraction Kit, Qiagen, Hilden, Germany) were cloned into the pGEM^®^-T Easy Vector (Promega, Madison, WI, USA) and confirmed by DNA sequencing. The plasmid was then digested with restriction enzymes (Figure 4), subcloned into the promoterless pEGFP-1 Vector (BD Biosciences Clontech, San Jose, CA, USA) or pGL3-Basic vector (Promega, Madison, WI, USA), and confirmed by sequencing. Truncated constructs were generated by PCR using the full-length sequence as a template; these are shown in Figure 5E. For the fluorescence assay, 0.5 μg pPSRP:EGFP construct was transiently transfected into 10^5^ CHSE-214 cells per well by electroporation (MicroPoration MP-100, Life Technologies, Carlsbad, CA, USA); cells were then stimulated with either saImon recombinant rIFN-α [45] (0.5 μg/mL) or IPNV (MOI = 1). For the luciferase assay, 0.5 μg pGL3-basic-PSRP construct and 10 ng *Renilla luciferase* plasmid (as an internal control) were transiently co-transfected into 10^5^ CHSE-214 cells per well by electroporation and then stimulated with either 0.5 μg/mL rIFN-α or IPNV (MOI = 1). The Dual Luciferase Assay Kit (Promega, Madison, WI, USA) was used to analyze PSR activation. All data are presented as the fold increase in relative luciferase activity (RLU) compared to the promoterless vector, and experiments were performed in triplicate. Statistical significance between the treatment and control groups was analyzed using Student’s *t* test (*p* < 0.05).

### 3.4. Morpholino Treatment

The morpholino utilized was obtained from Gene Tools, LLC (Philomath, OR, USA). The sequence was designed by Blossom Biotechnologies Inc. (Taipei, Taiwan) based on gene accession number EF067841, and the sequence was as follows: IRF-1-MO, 5'-TCATTCTCATCCTAgACACAggCAT-3' (3' labeling with FITC, fluorescein isothiocyanate). The control morpholino was CCTCTTACCTCAGTTACAATTTATA (Gene-Tools, Philomath, OR, USA). Morpholino oligos were solubilized in nuclease-free water at a concentration of 2 mM and diluted in nuclease-free water to 500 μM for use. We delivered 20 μL morphlino oligos with 6 μL Endo-Porter Gene Tools and 1 mL 10% serum medium in a 6-cm Petri dish, and the final concentrations of these were 10 and 6 μM, respectively. After 18 h of morpholino oligos treatment, we infected cells with IPNV and then performed immunoblotting and flow cytometry analysis.

### 3.5. WST-1 Assay for Quantification of Cell Viability

For cell viability assays, CHSE-214 cells were seeded in 24-well tissue culture plates and grown to partial confluence (80%). After IPNV infection (MOI = 1), cell viability was measured by a colorimetric assay that was based on the cleavage of the tetrazolium salt WST-1 (Roche, Mannheim, Germany) by using mitochondrial dehydrogenases. Each sample was harvested in 200 μL MEM with 20 μL WST-1 reagent. The optical densities (O.D.450 and O.D.690) of each sample were measured with a Bio-Rad 680 microplate reader (Bio-Rad, Hercules, CA, USA), and the data were calculated at the wavelengths 450 and 690 nm (Abs.450 minus Abs.690) and are presented as the means ± standard deviation (SD) of three repeats. Statistical significance between treatment and control groups was analyzed using Student’s *t* test (*p* < 0.05).

### 3.6. Flow Cytometric Analysis

CHSE-214 cells (1 × 10^5^) were stained for Annexin V and Propidium iodide (PI) (Annexin V-FLUOS Staining Kit, Roche, Mannheim, Germany) and then analyzed with a BD FACSCanto II flow cytometer (BD Biosciences, San Jose, CA, USA). Four groups were recognized and represented live, apoptotic, post-apoptotic necrotic and necrotic cells. The live cells were not stained with any dyes; the apoptotic cells were stained only with Annexin V on exposed phosphatidylserine (PS) (green); post-apoptotic cells were stained with both dyes (green and red); and the necrotic cells were stained only with Propidium iodide (PI) in the nucleus (red). Samples were then analyzed with BD FACS Diva Software (BD Biosciences, San Jose, CA, USA), and background correction was performed. The data are presented as the means ± SD of three repeats. Statistical significance between the treatment and control groups was analyzed using Student’s *t* test (*p* < 0.05).

### 3.7. Immunoblotting

CHSE-214 cells (1 × 10^5^) were cultured in a 6-cm Petri dish, subsequently incubated either with or without IRF-1 morpholino and then further infected with IPNV (MOI = 1) at 18 °C for 0, 4, 8, 12 and 24 h. At each time point, cells were collected and solubilized in 150 μL RIPA buffer containing 25 mM Tris–HCl (pH 7.6), 150 mM NaCl, 1% NP-40, 1% sodium deoxycholate, 0.1% SDS and complete protease inhibitor (Roche, Mannheim, Germany). The samples were then sonicated, boiled with sample buffer (Fermentas, Vilnius, Lithuania), subjected to SDS-polyacrylamide gel electrophoresis on denaturing 12% or 15% polyacrylamide gels (depending on protein size), transferred to polyvinylidene difluoride (PVDF) membranes, blocked overnight with 5% nonfat milk, and reacted with antibodies against PSR, β-actin, IRF-1, rIFN-α and IPNV whole proteins (Sigma, St. Louis, MO, USA and Santa Cruz, TX, USA). The membranes were rinsed in phosphate-buffered saline with Tween 20 (PBST), reacted with anti-rabbit, anti-mouse, anti-goat immunoglobulins conjugated to horseradish peroxidase (HRP) and developed with an enhanced chemiluminescence Western blot detection system Kit (Thermo, Vernon Hills, IL, USA).

### 3.8. Real-Time Quantitative PCR

The total RNA of CHSE-214 cells was harvested at each time point from a 6-cm Petri dish, isolated with 1 mL TRIzol reagent (Invitrogen, Carlsbad, CA, USA) and then extracted with 0.2 mL chloroform followed by 0.5 mL isopropanol and 1 mL ethanol; RNA was finally dissolved in 30 μL nuclease-free water. Sixty four nanograms of total RNA were used to quantify gene expression levels using a Roche Lightcycler^®^ 480 (Roche Applied Science, Mannheim, Germany). Real-time reverse transcription-polymerase chain reaction (RT-PCR) amplification for cDNA synthesis was performed with the High Capacity cDNA Archive Kit (ABI, Applied Biosystems, Grand Island, NY, USA) under the following conditions: 25 °C for 10 min, 37 °C for 2 h, and finally 85 °C for 5 s. The primers for quantitative PCR and RT-PCR were designed with Primer 3 (version 0.4.0, Whitehead Institute for Biomedical Researchproducer, Cambridge, MA, USA, [46]). Quantitative PCR using ABI Fast SYBR^®^ Green Master Mix was performed for 50 cycles with cycling conditions of 3 s at 95 °C and 30 s at 60 °C. The real-time PCR signals were analyzed in a multiplex format using Roche Lightcycler^®^ 480 software (Version 1.2.9.11; Roche Applied Science, Mannheim, Germany). The house keeping gene salmon beta-actin gene was used for normalization. The data are presented as the means ± SD in triplicate. Statistical significance between treatment and control groups was analyzed using Student’s *t* test (*p* < 0.05).

## 4. Conclusions

**Figure 9:**
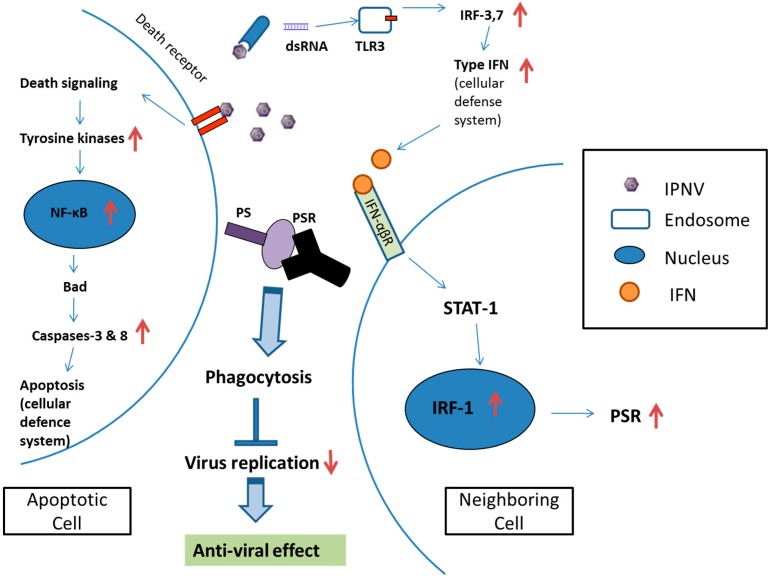
The cellular defense mechanisms induced by IPNV infection in CHSE-214 cells. After IPNV infection, two cellular defense mechanisms are induced: One is the apoptosis pathway, the other is the interferon pathway. Apoptosis is induced in some CHSE-214 cells via the caspase pathway and the transfer of PS from the inner to the outer cellular membrane. The exposed PS acts as an engulfment signal to induce PSR in neighboring cells via IRF-1 regulation. PSR is located on the surface of the neighboring cell and recognizes the exposed PS on the apoptotic cell. PSR-PS binding promotes the clearance of the apoptotic cell and inhibits viral replication to promote anti-viral effects. The up and down red arrows indicate the genes which were up-regulated or down-regulated by IPNV infection respectively.

The PSR gene expression is involved in IRF-1 regulation and also determines viral expression. Neighboring cells express PSR and promote the engulfment of apoptotic cells (Figure 5B). Therefore, these results suggests that PSR actually plays a crucial role in the clearance of apoptotic cells and promotes antiviral effects to facilitate the removal of the pathogen. Through PS-PSR binding, PSR initiates signaling events within the phagocytes that lead to the activation of the Rho GTPases Rac1, Cdc42 and WASp for cytoskeletal reorganization, allowing corpse internalization and signaling through this receptor. PSR also leads to the secretion of immunosuppressive cytokines such as TGF-β and possibly PGE2, IL-10, and platelet-activating factor (PAF) [6,10]. Moreover, the inhibition of IRF-1 causes a decrease in IPNV-induced apoptosis and delayed expression of PSR in IRF-1 knockdown cells during infection. Therefore, IRF-1 plays a dual role in regulating the induction of apoptosis and PSR during viral infection, and apoptosis precedes and subsequently promotes PSR expression. This is the first study to demonstrate the relationship between the apoptosis-related gene PSR and the immune-related transcription factor IRF-1, which exhibits a novel mechanism for PSR induction. IPNV can activate IRF-1, possibly through the type I IFN pathway activated by viral dsRNA, and can regulate PSR expression in CHSE-214 cells. The possible regulatory mechanism occurs via IRF-1 binding to the PSR promoter to activate PSR expression. In Figure 9, we describe a cellular defense mechanism following IPNV infection in CHSE-214 and provide an overview of the molecular mechanisms and immunological outcomes of apoptotic cell removal and further pathogen clearance. It has been suggested that IRF-1 is one of the principal host regulatory factors involved in cellular responses to combat viral infection via apoptosis and in the clearance of viruses from apoptotic cells via PSR induction. Taken together, IRF-1 potentially may play a key role in linking the immune response and apoptosis during viral infection, and the coexistence of phagocytic cells with virus-infected apoptotic cells could affect the extent of viral assembly. Our study should serve as a potential model to study viral pathogenesis and elucidate virus–host interactions.

## References

[1] Somersan S., Bhardwaj N. (2001). Tethering and tickling: A new role for the phosphatidylserine receptor. J. Cell Biol..

[2] Koninger J., Balaz P., Wagner M., Shi X., Cima I., Zimmermann A., di Sebastiano P., Buchler M.W., Friess H. (2005). Phosphatidylserine receptor in chronic pancreatitis: Evidence for a macrophage independent role. Ann. Surg..

[3] Hong J.R., Lin G.H., Lin C.J., Wang W.P., Lee C.C., Lin T.L., Wu J.L. (2004). Phosphatidylserine receptor is required for the engulfment of dead apoptotic cells and for normal embryonic development in zebrafish. Development.

[4] Walport M.J. (2001). Advances in immunology: Complement (second of two parts). N. Engl. J. Med..

[5] Schlegel R.A., Williamson P.P.S. (2007). To PS (phosphatidylserine)—Pertinent proteins in apoptotic cell clearance. Sci. STKE.

[6] Fadok V.A., Xue D., Henson P. (2001). If phosphatidylserine is the death knell, a new phosphatidylserine-specific receptor is the bellringer. Cell Death Differ..

[7] Zakharova L., Dadsetan S., Fomina A.F. (2009). Endogenous *Jmjd6* gene product is expressed at the cell surface and regulates phagocytosis in immature monocyte-like activated THP-1 cells. J. Cell. Physiol..

[8] Wyllie A.H., Kerr J.F., Currie A.R. (1980). Cell death: The significance of apoptosis. Int. Rev. Cytol..

[9] Elliott M.R., Ravichandran K.S. (2010). Clearance of apoptotic cells: Implications in health and disease. J. Cell Biol..

[10] Poon I.K., Hulett M.D., Parish C.R. (2010). Molecular mechanisms of late apoptotic/necrotic cell clearance. Cell Death Differ..

[11] Tamura T., Yanai H., Savitsky D., Taniguchi T. (2008). The IRF family transcription factors in immunity and oncogenesis. Ann. Rev. Immunol..

[12] Hong J.R., Huang L.J., Wu J.L. (2005). Aquatic birnavirus induces apoptosis through activated caspase-8 and -3 in a zebrafish cell line. J. Fish Dis..

[13] Chen P.C., Wu J.L., Her G.M., Hong J.R. (2010). Aquatic birnavirus induces necrotic cell death via the mitochondria-mediated caspase pathway. Fish Shellfish Immunol..

[14] Ravichandran K.S. (2003). “Recruitment signals” from apoptotic cells: Invitation to a quiet meal. Cell.

[15] Fujimoto I., Pan J., Takizawa T., Nakanishi Y. (2000). Virus clearance through apoptosis-dependent phagocytosis of influenza a virus-infected cells by macrophages. J. Virol..

[16] Dobos P. (1995). The molecular biology of infectious pancreatic necrosis virus (IPNV). Ann. Rev. Fish Dis..

[17] Rodriguez Saint-Jean S., Borrego J.J., Perez-Prieto S.I. (2003). Infectious pancreatic necrosis virus: Biology, pathogenesis, and diagnostic methods. Adv. Virus Res..

[18] Roberts R.J., Pearson M.D. (2005). Infectious pancreatic necrosis in atlantic salmon, salmo salar l. J. Fish Dis..

[19] Larsen R., Rokenes T.P., Robertsen B. (2004). Inhibition of infectious pancreatic necrosis virus replication by atlantic salmon Mx1 protein. J. Virol..

[20] Robertsen B. (2008). Expression of interferon and interferon-induced genes in salmonids in response to virus infection, interferon-inducing compounds and vaccination. Fish Shellfish Immunol..

[21] Hedrick R.P., Okamoto N., Sano T., Fryer J.L. (1983). Biochemical characterization of eel virus european. J. Gen. Virol..

[22] Samuel C.E. (2001). Antiviral actions of interferons. Clin. Microbiol. Rev..

[23] Collet B., Munro E.S., Gahlawat S., Acosta F., Garcia J., Roemelt C., Zou J., Secombes C.J., Ellis A.E. (2007). Infectious pancreatic necrosis virus suppresses type I interferon signalling in rainbow trout gonad cell line but not in Atlantic salmon macrophages. Fish Shellfish Immunol..

[24] Skjesol A., Aamo T., Hegseth M.N., Robertsen B., Jorgensen J.B. (2009). The interplay between infectious pancreatic necrosis virus (IPNV) and the ifn system: IFN signaling is inhibited by IPNV infection. Virus Res..

[25] Robertsen B. (2006). The interferon system of teleost fish. Fish Shellfish Immunol..

[26] Barnes B., Lubyova B., Pitha P.M. (2002). On the role of IRF in host defense. J. Interferon Cytokine Res..

[27] Kanazawa N., Kurosaki M., Sakamoto N., Enomoto N., Itsui Y., Yamashiro T., Tanabe Y., Maekawa S., Nakagawa M., Chen C.H. (2004). Regulation of Hepatitis C virus replication by interferon regulatory factor 1. J. Virol..

[28] Kroger A., Koster M., Schroeder K., Hauser H., Mueller P.P. (2002). Activities of IRF-1. J. Interferon Cytokine Res..

[29] Miyamoto M., Fujita T., Kimura Y., Maruyama M., Harada H., Sudo Y., Miyata T., Taniguchi T. (1988). Regulated expression of a gene encoding a nuclear factor, IRF-1, that specifically binds to *IFN-β* gene regulatory elements. Cell.

[30] Taniguchi T., Ogasawara K., Takaoka A., Tanaka N. (2001). IRF family of transcription factors as regulators of host defense. Annu. Rev. Immunol..

[31] Robertsen B., Bergan V., Rokenes T., Larsen R., Albuquerque A. (2003). Atlantic salmon interferon genes: Cloning, sequence analysis, expression, and biological activity. J. Interferon Cytokine Res..

[32] Jorgensen J.B., Johansen A., Hegseth M.N., Zou J., Robertsen B., Collet B., Secombes C.J. (2007). A recombinant CHSE-214 cell line expressing an Mx1 promoter-reporter system responds to both interferon type I and type II from salmonids and represents a versatile tool to study the IFN-system in teleost fish. Fish Shellfish Immunol..

[33] Hong J.R., Lin T.L., Hsu Y.L., Wu J.L. (1998). Apoptosis precedes necrosis of fish cell line with infectious pancreatic necrosis virus infection. Virology.

[34] Espinoza J.C., Kuznar J. (2002). Rapid simultaneous detection and quantitation of infectious pancreatic necrosis virus (IPNV). J. Virol. Methods.

[35] Battistini A. (2009). Interferon regulatory factors in hematopoietic cell differentiation and immune regulation. J. I Interferon Cytokine Res..

[36] Sun B.J., Robertsen B., Wang Z.Q., Bin L. (2009). Identification of an atlantic salmon IFN multigene cluster encoding three IFN subtypes with very different expression properties. Dev. Comp. Immunol..

[37] Taniguchi T., Takaoka A. (2002). The interferon-α/β system in antiviral responses: A multimodal machinery of gene regulation by the IRF family of transcription factors. Curr. Opin. Immunol..

[38] Scherbik S.V., Stockman B.M., Brinton M.A. (2007). Differential expression of interferon (IFN) regulatory factors and IFN-stimulated genes at early times after west nile virus infection of mouse embryo fibroblasts. J. Virol..

[39] Chen J.Z., Liu X.S. (2009). The role of interferon γ in regulation of CD4^+^ T-cells and its clinical implications. Cell. Immunol..

[40] Bergan V., Kileng O., Sun B.J., Robertsen B. (2010). Regulation and function of interferon regulatory factors of atlantic salmon. Mol. Immunol..

[41] Stang M.T., Armstrong M.J., Watson G.A., Sung K.Y., Liu Y., Ren B., Yim J.H. (2007). Interferon regulatory factor-1-induced apoptosis mediated by a ligand-independent fas-associated death domain pathway in breast cancer cells. Oncogene.

[42] Wu J.L., Chang C.Y., Hsu Y.L. (1987). Characteristics of an infectious pancreatic necrosis like virus isolated from japanese eel (*Anguilla japonica*). Bull. Inst. Zool. Acad. Sin..

[43] Yutaka A. TFSEARCH Search Result. http://www.cbrc.jp/htbin/nph-tfsearch.

[44] Ensembl Genome Browser Release 77 for *Danio rerio*. http://asia.ensembl.org/Danio_rerio/Search/Results?species=Danio_rerio;idx=;q=psr.

[45] Xu C., Guo T.C., Mutoloki S., Haugland O., Marjara I.S., Evensen O. (2010). α Interferon and not γ interferon inhibits salmonid alphavirus subtype 3 replication *in vitro*. J. Virol..

[46] Primer3 Input. http://frodo.wi.mit.edu/.

